# Editorial: Mechanisms underlying the interactions between stress and pain

**DOI:** 10.3389/fpain.2023.1285257

**Published:** 2023-09-11

**Authors:** Roxana Florea, Sarah D. Linnstaedt, Sandrine M. Géranton

**Affiliations:** ^1^ Department of Cell and Developmental Biology, University College London, London, United Kingdom; ^2^ Department of Anesthesiology, University of North Carolina at Chapel Hill, Chapel Hill, NC, United States

**Keywords:** stress, pain, vulnerability, patient stratification, affective state

**Editorial on the Research Topic** Mechanisms underlying the interactions between stress and pain

## Introduction

Chronic pain results from a complex interplay between biological, psychological, and socioeconomic factors. To date, numerous risk factors have been associated with the increased susceptibility to chronic pain, from mental health state and lifestyle, to gender and the presence of medical comorbidities, such as insomnia and cardiovascular diseases. Despite the identification of these risk factors, managing chronic pain, let alone preventing it, remains a major clinical challenge. This is because the specific circuits and mediators underlying the diverse components of the multidimensional experience of pain are not specific to the processing of nociceptive information and are often engaged in response to other bodily threats, including stress exposure. This prompts the need for clinical and preclinical studies that examine the multifaceted aspects of chronic pain and how these may overlap with other long-term conditions.

The Research Topic *Mechanisms Underlying the Interactions Between Stress and Pain* includes four research articles that highlight the need for patient stratification based on an individual's affective state and stress vulnerability for effective pain relief, while the two review articles present an overview of the current knowledge regarding the neurobiological mechanisms via which stress exacerbates pain in both clinical and pre-clinical settings.

## Overview of the articles

Pain is a salient stimulus that requires immediate attention. Cognitive distractions have long been recognised as powerful mediators of attentional-induced analgesia (1), and, increasingly, virtual reality (VR) applications are shown to be effective at attenuating acute pain such as dental and ischemic pains, burns and wounds (2, 3). However, the overall aim of these novel VR approaches would be to produce long-lasting benefits on pain control that would outlast the exposure to the VR environment. As such, a deeper understanding of the mechanisms underlying the management of pain by VR exposure is warranted. In their study, Barcatta et al., provide evidence for the need to *stratify* subjects based on their affective state to adapt the cognitive load of the VR interventions to achieve efficient analgesia. Healthy volunteers were exposed to a low and a high demand cognitive task while given cutaneous thermal stimulations and asked to report the moment they perceived the stimulation as painful during the two tasks. Contrary to their hypothesis, Barcatta et al., found that increasing the cognitive load did not result in hypoalgesia in all participants, but only benefited those with low emotional distress. This insight is important as it adds to the emerging idea that distraction is not the sole mechanism for VR-induced analgesia (4). Secondly, it emphasizes the need for tailored VR experience, that takes affective state into account for optimal therapeutic pain-relieving VR intervention.

Identifying individual risk factors that increase the likelihood of developing chronic pain is becoming increasingly recognised as the way forward to achieve effective pain control. Ferdousi et al., looked at the effect of stress vulnerability in a persistent inflammatory injury on pain- and anxiety-related behaviours and cognitive impairment. For this study, they used two different rat strains, Wistar-Kyoto (WKY) and Sprague-Dawley. The WKY rodents are an inbred strain found to exhibit a depressive- and anxiety-like phenotype in numerous behavioural tests, as well as enhanced pain-like behaviours following injuries, compared to Sprague-Dawley rats and various other outbred strains. Differences in mechanical sensitivity before and after pain induction were observed between the two strains, as well as in their affective and cognitive behaviours, emphasizing the translatability of using WKY rats in preclinical pain research to uncover the mechanisms underlying the pathophysiology of comorbid conditions such as anxiety vulnerability, social withdrawal, and cognitive deficits.

Stress is known to enhance pain sensitivity in both humans and animals. Individuals living with inflammatory painful conditions, such as irritable bowel syndrome (IBS) or rheumatoid arthritis, are particularly at risk of experiencing symptom fluctuations or disease flare ups depending on their distress levels. In addition, IBS patients are also at greater risk of having clinical comorbidities, such as temporomandibular disorders (TMDs) compared to the general population (5). Given the increased preponderance of comorbid IBS and temporomandibular disorders and the ability of stress to exacerbate both conditions, Mocci et al., conducted a transcriptomic analysis of peripheral and spinal tissues exploring to what extent the genetic signatures tiggered in the development of persistent pain following stress exposure are the same as those evoked by the presence of multiple comorbid pain conditions. Their study contributes to the identification of the system-wide collection of molecules and pathways that are specifically modulated during distinct traumatic conditions and provide disease signatures of chronic pain that could potentially serve as biomarkers.

The study conducted by Drazich et al., emphasizes the importance of understanding patients' chronic pain from the perspective of a complex interplay between biological, psychological, and sociological factors. African American women have an increased risk for experiencing comorbid pain and depressive symptoms compared to other racial/ethnic groups, and they are also more likely to be undertreated, if at all, for either or both conditions. The authors suggest that tailored interventions that take into account cultural differences and personal preferences are required for therapies to be effective in chronic pain and depression sufferers from similar demographics.

In their concise review, Bella et al., discuss recent pre-clinical studies that provide insights into the behavioural changes and mechanisms that underlie the development of chronic post-surgical pain following exposure to stress. According to IASP, the incidence of chronic post-surgical pain has been underestimated in the past, with current studies indicating an incidence of 20%–30% at 6–12 months after surgery (6). Psychological risk factors such as distress, catastrophising, and anxiety, represent one of the main risk factors for developing chronic post-surgical pain (7). The underlying biological mechanisms are, however, largely unknown. The review discusses the impact of different stressors on the post-surgical pain experience and summarizes the evidence for a role of the HPA axis, neuroimmune interactions, as well as the role of major central neurotransmitter system alterations seen in stress-induced increase and prolongation of post-surgical hypersensitivity. Given the increased preponderance of chronic pain in the female population (8), sex differences in the mechanisms underlying stress-induced hypersensitivity are also addressed.

When pain becomes long lasting, the emergence of comorbidities is imminent, if not pre-existing. From anxiety and stress, to sleep disturbances and substance misuse, such adverse experiences complicate disease prognosis and in a vicious cycle, the individual's wellbeing is significantly impacted. With a focus on chronic pain and stress, Schaffer et al., provide a comprehensive review of predisposing factors and psychological features common to both long-lasting conditions. An overview of overlapping neural circuits then follows with a final focus on the role of ventromedial prefrontal cortex (VmPFC) in the regulation of stress, pain, and substance (mis)use. VmPFC receives extensive projections from pain- and stress-encoding brain regions, and in turn is involved in top-down modulation of such states as well as addiction via reciprocal connections with nucleus accumbens (9–11). A feed-forward model is proposed by Schaffer et al., wherein chronic stress alter VmPFC related circuits that regulate pain, stress, and reward, thus dysfunctional VmPFC circuits represent a risk factor for the development of chronic pain.

## Conclusion

The articles in this Research Topic highlight the complexity of the pain experience and the influence of genetic and environmental factors, in particular stress vulnerability, on the transition to a chronic pain state. For patients, chronic pain can be a symptom to an underlying problem, or a distinct disease. As such, the current Research Topic emphasises the importance of patient stratification and tailored interventions for effective management of pain symptoms.

## References

[1] Villemure C Bushnell CM. Cognitive modulation of pain: how do attention and emotion influence pain processing? Pain. (2002) 95(3):195–9. 10.1016/S0304-3959(02)00007-6 11839418

[2] Mallari B Spaeth EK Goh H Boyd BS. Virtual reality as an analgesic for acute and chronic pain in adults: a systematic review and meta-analysis. J Pain Res. (2019) 12:2053–85. 10.2147/JPR.S200498 31308733 PMC6613199

[3] Trost Z France C Anam M Shum C. Virtual reality approaches to pain: toward a state of the science. Pain. (2021) 162(2):325–31. 10.1097/j.pain.0000000000002060 32868750

[4] Wang X Shi Y Zhang B Chiang Y. The influence of forest resting environments on stress using virtual reality. Int J Environ Res Public Health. (2019) 16(18):3263. 10.3390/ijerph16183263 31491931 PMC6765889

[5] Gallotta S Bruno V Catapano S Mobilio N Ciacci C Iovino P. High risk of temporomandibular disorder in irritable bowel syndrome: is there a correlation with greater illness severity? World J Gastroenterol. (2017) 23(1):103–9. 10.3748/wjg.v23.i1.103 28104985 PMC5221272

[6] Schug SA Lavand’homme P Barke A Korwisi B Rief W Treede RD The IASP classification of chronic pain for ICD-11: chronic postsurgical or posttraumatic pain. Pain. (2019) 160(1):45–52. 10.1097/j.pain.0000000000001413 30586070

[7] Rosenberger DC Pogatzki-Zahn EM. Chronic post-surgical pain – update on incidence, risk factors and preventive treatment options. BJA Educ. (2022) 22(5):190–6. 10.1016/j.bjae.2021.11.008 35496645 PMC9039436

[8] Mills SEE Nicolson KP Smith BH. Chronic pain: a review of its epidemiology and associated factors in population-based studies. Br J Anaesth. (2019) 123(2):e273–83. 10.1016/j.bja.2019.03.023 31079836 PMC6676152

[9] Goldstein RZ Volkow ND. Dysfunction of the prefrontal cortex in addiction: neuroimaging findings and clinical implications. Nat Rev Neurosci. (2011) 12(11):652–69. 10.1038/nrn3119 22011681 PMC3462342

[10] McEwen BS. Physiology and neurobiology of stress and adaptation: central role of the brain. Physiol Rev. (2007) 87(3):873–904. 10.1152/physrev.00041.2006 17615391

[11] Ong WY Stohler CS Herr DR. Role of the prefrontal cortex in pain processing. Mol Neurobiol. (2019) 56(2):1137–66. 10.1007/s12035-018-1130-9 29876878 PMC6400876

